# Supplementary material for: Childhood appendectomy and subsequent psychiatric illness
Source: PLOS Ment Health. 2025 Jan 16;2(1):e0000219. doi: 10.1371/journal.pmen.0000219 (PMC12798240; doi:10.1371/journal.pmen.0000219)
Supplement: S1 Table — (DOCX) [file pmen.0000219.s001.docx]

**S1 Table.** ATC codes for drugs affecting the nervous system with corresponding ICD-10 codes

| **ATC** | **ICD-10** |
| --- | --- |
| N05B Anxiolytics  N05C Hypnotics and sedatives  N07B Drugs used in addictive disorders | F10-F19 Mental and behavioural disorders due to psychoactive substance use  F40-48 Neurotic, stress-related and somatoform disorders |
| N06A Antidepressants  N05AN (Lithium) | F30-39 Mood (affective) disorders |
| N05A Antipsychotics minus N05AN (Lithium) | F20-29 Schizophrenia, schizotypal and delusional disorders |
| N06B Psychostimulants, agents used for ADHD and nootropics | F84 Persvasive developmental disorders (autism, Aspberger syndrome etc)  F90-98 Behavioural and emotional disorders with onset usually occurring in childhood and adolescents |
|  | F50 Eating disorders |
