# Supplementary material for: Childhood appendectomy and subsequent psychiatric illness
Source: PLOS Ment Health. 2025 Jan 16;2(1):e0000219. doi: 10.1371/journal.pmen.0000219 (PMC12798240; doi:10.1371/journal.pmen.0000219)
Supplement: Attachment — Submitted filename: Response to reviewer (2).docx [file pmen.0000219.s002.docx]

Dear reviewer.

We are thankful for the insightful comments and believe the revision enriches and improves the manuscript overall.

___________________________________________________________________________

\# Comment by reviewer: One minor critic-why aren't any considerations for the medication dosages (i.e., NSAID, PPI, Antibiotics dosages in association with thereafter psychiatric illnesses) in assessing the risks of psychiatric illnesses? Would you add the dosage-dependent relationships analysis for the manuscript?

>> Reply: thank you for your time to review our work and the constructive feedback. We agree that indeed it would be interesting to add a dosage-dependent relationship analysis to explore this association between drugs use and psychiatric outcomes, as we did find strong (dose-response) effects of antibiotics in several of our previous association studies. Yet, in this project, drugs were confounding factors and not our main exposure, and we therefore did not explore this drug-outcome association in more depth.

We also have limited power for further sub-analyses, particularly for the non-antibiotic drugs, but it does indeed looks worthwhile to explore in more depth in a follow-up study including the whole Swedish population with appendicitis/appendectomy, as we now only had a subpopulation from the Uppsala region yet with detailed clinical information (more detailed than when we would only use the Swedish Health Registries). We did add this in the discussion as follows (page 12, row 234) “*Due to limited power and as the drugs were not the main exposure, we did not explore dose-response effects, yet it appears important to assess this potential association in more depth in further research, particularly for antibiotics. “*
