# Supplementary material for: Childhood appendectomy and subsequent psychiatric illness
Source: PLOS Ment Health. 2025 Jan 16;2(1):e0000219. doi: 10.1371/journal.pmen.0000219 (PMC12798240; doi:10.1371/journal.pmen.0000219)
Supplement: Attachment — Submitted filename: Response to reviewers.docx [file pmen.0000219.s003.docx]

REBUTTAL LETTER

Dear reviewer.

Thank you for your time to review our work and the constructive feedback. We are thankful for the insightful comments and believe the revision enriches and improves the manuscript overall.

___________________________________________________________________________

\# Comment by reviewer: I read with interest the manuscript entitled "Childhood appendectomy and subsequent psychiatric illness"
The introduction is comprehensive with a clear statement of the aim of the study at the end.

For appendectomy patients, it would be important to divide them into uncomplicated and complicated forms of appendicitis because there is a possibility that the course of treatment of complicated, especially perforated forms of appendicitis, can be a significant confounder.

>> Reply: Indeed, this is an important point raised. The two types of appendicitis differ in their treatment as a complicated appendicitis is postoperatively treated with broad-spectrum antibiotics until clinic and laboratory findings are improved. Even though this was not the main research question in our study we made a sub analysis using Cox regression, where we divided appendicitis in a complicated versus an uncomplicated group. In the results part of the manuscript page 10, line 192-194, we have written*: ”The severity of inflammation of the appendix, complicated disease (gangrenous or perforated), was not associated with an increased risk of psychiatric illness HR 0.86 (95% CI 0.67-1.10; p-value 0.222)”.*

To further address this in the discussion we have on page 12, line 234-250, written: “*In our study antibiotics were prescribed in 63.1% among cases after the appendectomy, more than double than among controls (24.5%). A relatively high proportion of these prescriptions were explained by complicated appendicitis treated with broad-spectrum antibiotics after the appendectomy. Yet, in our study we did not find an increased risk of psychiatric illness after complicated appendicitis compared to uncomplicated appendicitis. Due to limited power and as the drugs were not the main exposure, we did not explore dose-response effects, yet it appears important to assess this potential association in more depth in further research, particularly for antibiotics. Further, when attempting to control for use of antibiotics and psychiatric illness, the use of antibiotics within two years after appendectomy gave an increased HR for behavioural, emotional and affective disorders regardless of being a case or a control. For the other drugs affecting the microbial composition, PPI and NSAID, we did not see this effect. Whether the increased risk of psychiatric illness was associated with the removal of the appendix, the exposure to antibiotics, the targeted infection or a combination of these remains unknown.”*

Even though we did not find a difference in the Cox regression analysis comparing complicated and uncomplicated appendicitis with the risk of psychiatric illness there might be a difference if we would compare different sub groups of psychiatric diagnoses. However, due to limited power we have not made this analysis. To address this we have written the discussion part on page 13, line 238-242 and also added the sentence on line 242-243 (written in red): “*Yet, in our study we did not find an increased risk of psychiatric illness after complicated appendicitis compared to uncomplicated appendicitis. Due to limited power and as the drugs were not the main exposure, we did not explore dose-response effects, yet it appears important to assess this potential association in more depth in further research, particularly for antibiotics. Neither did we investigate if complicated appendicitis affected specific subgroups of psychiatric illness* *because of the limited power when subdividing into subgroups.*

Can you guarantee that all patients who underwent appendectomy up to the age of 15 did not have other associated diseases during the specified period?

>> Reply: No, we can not guarantee this, neither can we guarantee that the matched controls had any associated diseases. Through the matched design, the effect of known and unknown confounders should be limited. The cohort of patients that underwent appendectomy before the age of 15 was used in a previous study where we compared the risk of small bowel obstruction after laparoscopic and open appendectomy. It was when conducting that study data from the patients charts were retrieved. We only extracted data concerning the appendectomy itself and health care visits related to possible complications of the appendectomy. We did not extract other data from the patients’ charts. All data on psychiatric illness in our study come from Swedish National Registers.

Please add to the statistical analysis section the way you tested the normality of the data distribution.

>> Reply: The statistical methods utilized in the analysis, Poisson regression and Cox regression, are not based on the normal distribution assumption. However, the Shoenfeld residuals was used to check the proportional hazard assumption in the Cox regression model. Descriptive statistics for continuous variables were summarized with median and interquartile range, which are robust measures for central tendency and scale and are representative measures even if the data is not normally distributed. Since the analysis was not reliant on data being normally distributed, testing for if the data followed a normal data distribution was not performed.

We have added the following sentence in the methods part of the manuscript. Page 7 lines 148-150: *“The proportional hazards assumption was checked by using statistical tests and graphical diagnostics based on the scaled Schoenfeld residuals.”*

It is unclear, in relation to table 1, whether the control group was exposed to appendectomy!?

>> Reply: Thank you for this comment. We will clarify this by changing the names of the groups throughout the manuscript including tables to

1) Exposed (those who has undergone appendectomy)

2) Non-exposed (matched individuals who has not undergone previous appendectomy)

In Table 1, p-values should be listed and next to them, indicate with a superscript which statistical test was used.

>> Reply: We have added this, please see Table 1.

You stated the following "Individuals with a psychiatric diagnosis or at least two dispensations of drugs affecting the nervous system before the date of appendectomy were excluded from further analysis." In relation to the table, the statement is incorrect.

>> Reply: Indeed, in the table there is a row saying “age group at time of appendectomy” and also ”age at time of appendectomy”. Of course this is confusing and incorrect as only the study cohort was exposed to appendectomy. We have made following changes to table 1 to clarify, please see below.

Also, in the methodology section we have now added a new term “index”, page 5, lines 93-95: *“The term index was used for the date of appendectomy for the exposed individuals. For the matched non-exposed individuals, index was the date of appendectomy for their matched exposed individual. “*

| **Variable** | **Non-exposed (N=3760)** | **Exposed (N=752)** | **P-value** |
| --- | --- | --- | --- |
| Follow-up time in years, (median, IQR) | 15.5 (11.9-18.9) | 15.5 (11.9-18.9) | 1.000 |
| Age at time of index in years, (median, IQR) | 11 (9-13) | 11 (9-13) | 0.9682 |
| Age groups at time of index |  |  |  |
| Preschool (1-5 years) | 220 (5.9) | 39 (5.2) | 0.6963 |
| Elementary school (6-9 years) | 962 (25.6) | 193 (25.7) |  |
| Middle school (10-12 years) | 1444 (38.4) | 286 (38.0) |  |
| High school (13-15 years) | 1134 (30.2) | 234 (31.1) |  |
| Sex |  |  |  |
| Male | 2280 (60.6) | 456 (60.6) | 1.0000 |
| **Psychiatric illness** |  |  |  |
| No diagnosis | 2421 (64.4) | 452 (60.1) | 0.1053 |
| Diagnosis before index* | 156 (4.1) | 34 (4.5) |  |
| Diagnosis within two years after index | 97 (2.6) | 27 (3.6) |  |
| Diagnosis two years after index | 1086 (28.9) | 239 (31.8) |  |
| **Diagnosis identified from** |  |  |  |
| No diagnosis | 2421 (64.4) | 452 (60.1) | 0.0662 |
| From NPR | 347 (9.2) | 66 (8.8) |  |
| From PDR | 280 (7.4) | 68 (9.0) |  |
| From NPR and PDR combined | 712 (18.9) | 166 (22.1) |  |
| **Prescribed** **drugs** ** | **Non-exposed** **(N=1870)** | **Exposed** **(N=374)** |  |
| Proton pump inhibitors | 25 (1.3) | 9 (2.4) | 0.1889 |
| Non steroidal anti-inflammatory drugs | 53 (2.8) | 17 (4.5) | 0.1153 |
| Antibiotics | 458 (24.5) | 236 (63.1) | <0.0001 |

Values in parentheses are percentages unless otherwise indicated.

P-values for categorical variables are from chi-square test. P-values for numerical variables (follow-up time, age at time of index in years) are from Mann-Whitney U-test.

* Individuals with a psychiatric diagnosis registered in NPR, or at least two dispensations of drugs affecting the nervous system in SDR before index.

** Data on prescribed drugs from 1 July 2005 and onwards, from index to two years onwards.

The discussion largely recognizes the many limitations of the study, which significantly reduce its significance of the study, and the conclusion is written rather boldly considering the numerous limitations.
In conclusion, considering the methodology and results, it is unclear who is the control group and who is the target group? Who is appendectomized and who is not? It's completely unclear.

>> Response: In the manuscript we have made a revision in the text addressing the strengths and limitations of the study, page 14, lines 277-2 .

*“One of the major strengths of the study are that the medical records were individually reviewed for the cases with detailed information confirming appendicitis and the subsequent treatment and data not only data based on registers. By adding the SDR to PDR, the study also includes individuals treated only within the primary care. With the definition of psychiatric illness as at least two prescriptions of drugs to classify as a positive result we aimed to increase the accuracy of drugs being a proxy for a positive case of psychiatric illness. Also the matching of individuals by county reduced the risk of systemic variation in clinical practice and healthcare access.*

*One of the major limitations is that we do not have data on familiar history of psychiatric illness or other socioeconomic factors that could affect the outcome of both psychiatric illness and health seeking behavior. Further, data from PDR is limited to the start of the register in 2005 which leaves five years of this study without this extra data. Also, our definition of psychiatric illness will exclude individuals treated by talk therapy alone in the primary care.”*

We have also rephrased the last part of the discussion pages 14-15, lines 294-308:

According to data from the World Health Organization around 20% of the world´s children and adolescents have a mental health condition, depression and anxiety being two of the most common, and the second leading cause of death among 15–29-year-olds. Psychiatric illness can affect many areas in life with a loss of function impairing the ability to participate and contribute in the community (30). As appendicitis is the most common surgical emergency during childhood, further investigation on the effect of childhood appendectomy on mental health is warranted. The findings from this and previous studies on the topic should be taken into consideration when making new guidelines on how to treat uncomplicated appendicitis in children. Information about this possible consequence after appendectomy is important for the operating surgeon and the caregivers of the child to enhance the prevention, early detection and treatment of psychiatric illness.
